# Divergent *Alphacoronavirus* Lineages Identified in Alabama Bats, USA

**DOI:** 10.3390/v18080887

**Published:** 2026-08-12

**Authors:** Subarna Barua, Charles Rupprecht, Daniel Felipe Barrantes Murillo, Chengming Wang

**Affiliations:** 1 Department of Pathobiology, College of Veterinary Medicine, Auburn University, Auburn, AL 36849, USA; szb0116@auburn.edu (S.B.); charleserupprechtii@gmail.com (C.R.); 2 College of Forestry, Wildlife & Environment, Auburn University, Auburn, AL 36849, USA; 3 Department of Veterinary Pathobiology, College of Veterinary Medicine, Oklahoma State University, Stillwater, OK 74076, USA; dabarra@okstate.edu

**Keywords:** bat coronavirus, *Alphacoronavirus*, *Eptesicus fuscus*, surveillance

## Abstract

Bats are crucial reservoirs for coronaviruses, yet surveillance in the southeastern United States remains limited. Using carcasses submitted from public health surveillance for rabies, we screened 170 non-rabid bat carcasses from Alabama using a tiered real-time PCR framework targeting broad *Alpha*- and *Betacoronavirus*, alongside specific *Sarbecovirus* and *Merbecovirus* lineages. Overall, coronavirus prevalence was 3.5% (6/170), with all positives resolving as *Alphacoronavirus*. Detections spanned six counties and three bat taxa: *Tadarida brasiliensis*, *Myotis* spp., and *Eptesicus fuscus*, with the latter accounting for most cases (4/6; 66.7%). Sequence and phylogenetic analyses revealed two distinct evolutionary lineages. One isolate, ID-51 (*E. fuscus*), exhibited relative conservation, sharing 95.3% nucleotide and 93.0% amino acid identity with known references, forming a distinct sibling branch. Conversely, a highly divergent cluster of five isolates (ID-06, -145, -147, -169, -175) displayed remarkably low nucleotide similarities (83.7%–84.3%) to GenBank references despite maintaining high amino acid conservation (98.3%–100%). Topological shifts between nucleotide and amino acid phylogenies demonstrated genetic variation within this novel cluster. These findings underscore a previously uncharacterized genetic diversity of coronaviruses in regional US wildlife, highlighting the necessity of expanded local genomic surveillance.

## 1. Introduction

Bats (order Chiroptera) represent one of the most species-rich mammalian orders, comprising over 1500 species globally that exhibit an unparalleled diversity of ecological and physiological adaptations [1,2]. This remarkable diversity, combined with life-history traits such as exceptional longevity, gregarious roosting behaviors, and the capacity for sustained flight, has positioned bats as natural reservoirs for a disproportionate number of zoonotic viruses relative to other mammalian groups [3]. Notably, these include high-consequence pathogens, such as SARS-like coronaviruses, Marburg hemorrhagic fevers, Nipah and Hendra virus infections, and rabies-causing lyssaviruses [2,4,5].

Despite harboring substantial viral diversity, bats rarely manifest clinical disease from many of these infections. This profound viral tolerance to several pathogens is increasingly attributed to unique immunological adaptations, including dampened STING-pathway signaling, reduced NLRP3 inflammasome activity, and the constitutive expression of type I interferons in select species [6,7,8,9,10,11]. However, this capacity for asymptomatic viral tolerance raises significant public health concerns regarding potential cross-species spillovers. Human encroachment, habitat fragmentation, and anthropogenic land-use changes increasingly drive interactions among bats, domestic animals, and humans [12,13]. Balancing this dual reality, in which bats provide indispensable ecological services yet represent a reservoir of public health concern, demands a One Health approach that pairs robust wildlife conservation with targeted, evidence-based pathogen surveillance using highly sensitive molecular methods.

Coronaviruses (family *Coronaviridae*) are classified into four genera, of which *Alphacoronavirus* and *Betacoronavirus* are known to infect mammals, including bats and humans. Alphacoronaviruses include several important human and animal pathogens, among them HCoV-229E and HCoV-NL63, which cause mild to moderate respiratory illness in humans, as well as feline infectious peritonitis virus (FIPV), canine coronavirus (CCoV), porcine epidemic diarrhea virus (PEDV), and transmissible gastroenteritis virus (TGEV) in domestic and livestock animals [14]. A broad diversity of bat alphacoronaviruses, including those previously detected in *Eptesicus*, *Tadarida*, and *Myotis* spp. [15,16] are thought to represent the ancestral reservoir from which mammalian alphacoronaviruses have evolved [17]. Within *Betacoronavirus*, the subgenus *Sarbecovirus* encompasses SARS-CoV and SARS-CoV-2, responsible for the 2003 SARS epidemic and the COVID-19 pandemic, respectively, while *Merbecovirus* includes MERS-CoV and related bat coronaviruses [15,18,19]. All three major pathogens are of bat origin, underscoring the vital importance of molecular surveillance targeting these specific subgenera. Although bats are primary reservoirs for alpha- and betacoronaviruses, mammalian infections are not strictly restricted to these lineages. Gammacoronaviruses (e.g., Beluga whale coronavirus SW1) and deltacoronaviruses (e.g., porcine and human-infecting deltacoronaviruses) demonstrate that both genera are capable of crossing host barriers into mammals [14,20,21].

Despite the profound global impact of these viruses, coronavirus surveillance in North American bats remains comparatively sparse relative to intensive efforts in Asia and Europe. The earliest evidence of coronaviruses in Western Hemisphere bats was documented in the Rocky Mountain region, revealing a viral prevalence of 17% in *Eptesicus fuscus* and 50% in *Myotis occultus* [15,18,19], which was followed by the identification of multiple alphacoronaviruses in eastern U.S. bat species [22]. Across the Americas, alphacoronaviruses predominate, accounting for approximately 89.4% of all documented bat coronavirus detections, with betacoronaviruses comprising the remainder [23]. Nevertheless, systematic, large-scale surveillance across North America remains limited, leaving substantial geographic gaps in our understanding of viral distribution, particularly within the southeastern U.S.

The gastrointestinal (GI) tract serves as a major site of replication and viral shedding for many bat-associated coronaviruses [24,25]. For instance, the MERS-CoV receptor, dipeptidyl peptidase 4 (DPP4), is predominantly expressed in the intestinal epithelium of insectivorous bats, supporting the GI tract as a key site of localized infection and indicating that fecal-oral transmission plays a fundamental role in bat coronavirus ecology [26]. Furthermore, experimental infections have demonstrated prolonged fecal shedding of coronaviruses [23,27], and field studies consistently detect persistent viral RNA in intestinal tissues [28]. Consequently, intestinal tissue is an optimal target matrix for molecular surveillance, maximizing the likelihood of detecting actively circulating bat coronaviruses.

To address current geographical and taxonomic gaps, we established and validated a tiered, three-assay SYBR Green real-time PCR platform. This system uniquely combines broad-spectrum *Alpha*-/ and *Betacoronavirus* detection with targeted, high-specificity *Sarbecovirus*- and *Merbecovirus*-screening panels. This multifaceted approach was developed because a single, universal assay cannot reliably accommodate the extensive genetic polymorphisms characteristic of circulating coronaviruses, making pan-coronavirus protocols prone to false negatives due to primer–template mismatches [29,30,31]. By applying this tiered molecular screening framework to intestinal tissues harvested from bats in Alabama, this study provides critical baseline data on regional coronavirus prevalence and evolutionary diversity, ultimately supporting future surveillance efforts, zoonotic risk assessments, and One Health pandemic preparedness in an understudied region of the southeastern U.S.

## 2. Materials and Methods

### 2.1. Bats

A total of 170 non-rabid bat carcasses, submitted by the public to the Alabama Department of Public Health between 2023 and 2025 and as described in reference [32] were included in this study. Bat carcasses were previously identified to species level using a combined molecular and morphological approach [32].

Frozen bats underwent necropsy using sterile, single-use disposable necropsy kits for each animal to prevent cross-contamination between specimens. A ventral midline incision was made to expose the abdominal cavity, removing the gastrointestinal contents and collecting 40 mg of the large intestine. The tissue was homogenized in 300 µL of PBS using zirconia beads and a Precellys 24 tissue homogenizer (Bertin Technologies, Paris, France). Nucleic acids were extracted from 200 µL of homogenate using the IndiMag 2 automated magnetic-bead system (INDICAL Inc., Orlanda, FL, USA) according to the manufacturer’s instructions, and RNA was eluted in 100 µL of elution buffer and stored at −80 °C until analysis.

### 2.2. PCRs

To capture the extensive and evolving genetic diversity of bat-associated coronaviruses, strategic efforts were undertaken to design a robust molecular screening framework. Given the high degree of genetic polymorphism inherent in coronaviruses, a single universal PCR assay cannot reliably amplify all viral types across different genera. Therefore, after systematically evaluating multiple target genes across diverse coronavirus groups, a complementary three-assay real-time PCR approach was developed. To inform primer design, representative coronavirus nucleotide sequences reflecting known global diversity were retrieved from the National Center for Biotechnology Information (NCBI) GenBank database. This dataset included members of the subgenera *Sarbecovirus* (e.g., DQ022305, KF367457.1, KC881005.1) and *Merbecovirus* (e.g., EF065505, KC869678), as well as representative *Alpha*- and *Betacoronavirus* outside these groups (e.g., EF065513, HM211100, KJ473822, KY073747, EF203067, MG557844, and HQ728480) (Table 1, Figures S1–S3). Multiple sequence alignments were performed using Vector NTI to identify conserved genomic regions suitable for primer design. Based on phylogenetic relationships and sequence conservation, the target sequences were classified into three distinct screening panels: (i) a broad-spectrum panel targeting diverse *Alpha*- and *Betacoronavirus* (ii) *Sarbecovirus*, and (iii) *Merbecovirus*. Conserved regions identified within each group were subsequently selected as targets for the development of group-specific SYBR Green real-time PCR assays (Table 1; Supplementary Figures S1–S3). Initial primer design was performed using Vector NTI and rigorously assessed via BLAST analysis against the NCBI nucleotide database to ensure specificity and breadth of coverage.

Thermal cycling for differential dermatophyte qPCR was performed using a LightCycler^®^ 480 II real-time PCR platform (Roche Diagnostics, Indianapolis, IN, USA). For the SYBR assay, the high-stringency phase comprised 6 cycles of 10 s at 95 °C, 10 s at 70 °C, and 10 s at 72 °C; 9 cycles of 10 s at 95 °C, 10 s at 68 °C, and 10 s at 72 °C; and 3 cycles of 10 s at 95 °C, 10 s at 66 °C, and 10 s at 72 °C. This was followed by 30 relaxed-stringency fluorescence acquisition cycles for SYBR Green of 10 s at 95 °C, 10 s at 57 °C, and 10 s at 72 °C [33]. Reactions were performed in 10 µL of master mixture and 10 µL of sample nucleic acids or standards. Each 20 µL reaction contained 2.0 U Platinum^®^Taq DNA polymerase (Invitrogen, Carlsbad, CA, USA) and 0.0213 U ThermoScript™ reverse transcriptase (Invitrogen). The primers were used at 1 µM [34].

Synthetic double-stranded DNA fragments (gBlocks; Integrated DNA Technologies, Coralville, IA, USA) containing the corresponding primer-binding sites and target amplicon sequences were designed as positive controls for assay validation and sensitivity testing. Based on the molecular weight of each gBlock, 10-fold serial dilutions ranging from 10^4^ to 10^0^ copies per 10 µL reaction were prepared in triplicate to determine the limit of detection. In addition, genomic RNA of two SARS-CoV-2 viruses from the American Type Culture Collection (ATCC) (2019-nCOV/USA-WA1/2020; 201/501Y.V1) served as controls and quantitative standards in the *Sarbecovirus* assay. The *Sarbecovirus* primer sequences exhibited 100% sequence identity with SARS-CoV-2 genomes deposited in GenBank (OY664737, PQ594584, OX129731, OW740124).

The PCR products generated from positive bat samples were submitted to ELIM Biopharmaceuticals for bidirectional Sanger sequencing. The resulting nucleotide sequences were analyzed using the NCBI BLASTn tool to confirm the coronavirus group.

Molecular phylogenetic analysis of the detected coronavirus RNA sequences was conducted using the Maximum Likelihood (ML) method with the Tamura-Nei substitution model, as implemented in MEGA12 [35]. The robustness of the inferred topology was evaluated by bootstrap analysis with 1000 replicates, with support values displayed at each node, and evolutionary distances are expressed as the number of nucleotide substitutions per site as indicated by the scale bar.

## 3. Results

The three SYBR Green real-time PCR assays, designed to target *Sarbecovirus*, *Merbecovirus*, and a broad spectrum of *Alpha*- and *Betacoronavirus*, were validated using synthetic gBlock controls. All assays demonstrated consistent analytical sensitivity, with a limit of detection of 10 copies per reaction. The *Sarbecovirus*-specific assay was also validated by successfully amplifying RNA from two ATCC SARS-CoV-2 reference strains used as positive controls.

The 170 bat carcasses included in this study represented nine bat species across six genera. *E. fuscus* (48.8%) was the predominant species in the study population, followed by *Tadarida brasiliensis* (15.9%), *Nycticeius humeralis* (14.7%), *Lasiurus borealis* (11.2%), *Lasiurus seminolus* (2.9%), *M. dominicensis* (2.9%), *M. austroriparius* (1.8%), *Pipistrellus subflavus* (1.2%) and *Lasiurus ega* (0.6%). Coronavirus RNA was detected in 6 of the 170 specimens (3.5%). All positive samples were identified exclusively via the broad *Alpha*-/*Betacoronavirus* assay. No samples tested positive using the *Sarbecovirus*- or *Merbecovirus*-specific assays.

Positive detections occurred across three bat taxa and six Alabama counties (Coosa, Cherokee, Etowah, Madison, Jefferson, and Lauderdale). Most positive cases (66.7%; 4/6) were found in *E. fuscus* (the big brown bat), while *Tadarida brasiliensis* (the Brazilian free-tailed bat) and *Myotis* spp. (the mouse-eared bats) each accounted for a single positive case (Table 2). These findings demonstrate the circulation of coronaviruses among diverse bat species in Alabama, suggesting that *E. fuscus* may represent a primary host within the sampled populations.

**Figure 1:**
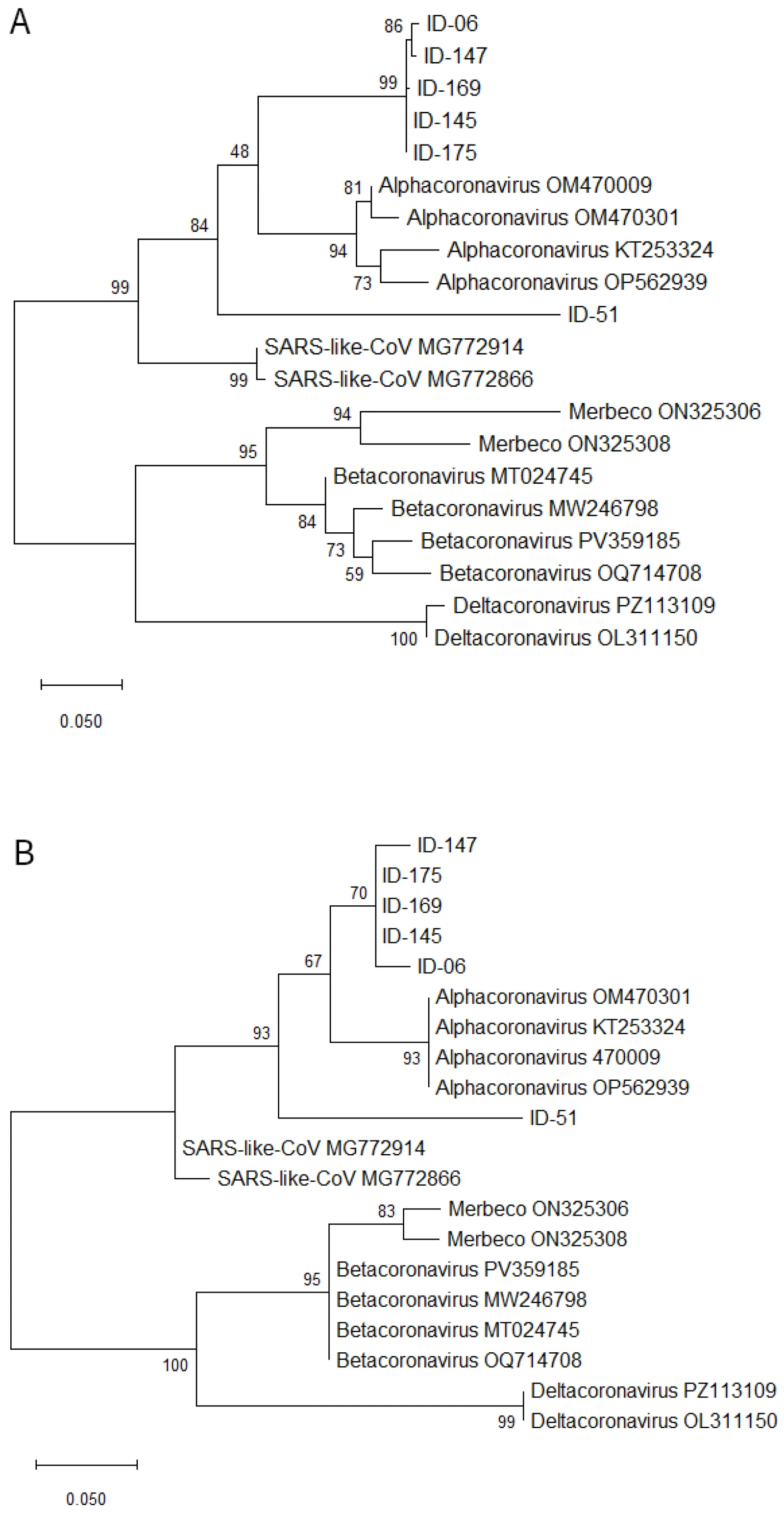
Phylogenetic relationships of *Alphacoronavirus* isolates detected in Alabama bats. Molecular phylogenetic trees were inferred using the Maximum Likelihood method [35] in MEGA12 based on the alignment of 171 nucleotides (**A**) and 57 translated amino acids (**B**) of the conserved replicase region. The nucleotide-based phylogenetic analysis was performed using a 171 bp fragment; therefore, some branches exhibit relatively low bootstrap support due to the limited phylogenetic information in the short amplicon. Bootstrap values (1000 replicates) are shown at the nodes, and the scale bars indicate genetic distance (substitutions per site). The analysis shows all six positive samples clustering within the *Alphacoronavirus* genus, remaining distinct from *Betacoronavirus*, *Sarbecovirus*, and *Merbecovirus* lineages. Two separate evolutionary patterns are revealed: a distinct sibling variant represented by isolate ID-51, sharing 95.1% nucleotide (92.9% amino acid) identity with reference strains, and a highly divergent, tightly clustered lineage comprising isolates ID-06, ID-145, ID-147, ID-175, and ID-169. The topological shift in the ID-06 and ID-147 variants between the nucleotide and amino acid trees indicates a high saturation of synonymous mutations or distinct evolutionary pressures acting upon this novel cluster.

Sanger sequencing confirmed all six amplicons as bat coronaviruses (Table 2). Subsequent BLAST analysis at 100% query coverage, combined with phylogenetic reconstruction based on a 171-nucleotide fragment of the conserved replicase (RdRp) region (Figure 1A) and its corresponding 57-amino acid translated sequence (Figure 1B), revealed two distinct genetic patterns among the six isolates. One isolate, ID-51, detected in *E. fuscus* from Cherokee County, clustered closely with known references. The sample shared 95.3%–93.0% nucleotide identity and up to 93.0% amino acid identity with existing database entries (e.g., UUA807797). The Synonymous Divergent Cluster (ID-06, ID-145, ID-147, ID-169, ID-175) exhibited a profound contrast between nucleotide and amino acid sequences. Nucleotide identities were uniformly low, ranging from 83.7%–84.3% down to 78.5%–79.2% against top GenBank hits (Figure 1A). Conversely, at the amino acid level, isolates ID-145, ID-169, and ID-175 exhibited 100% identity to reference ASL05100, while ID-06 and ID-147 maintained 98.3% identity (Figure 1B).

This disparity highlights a high saturation of synonymous mutations within the cluster. It also helps to explain the topological shifts observed between the nucleotide and amino acid phylogenetic trees (Figure 1). While these five samples represent highly divergent nucleotide lineages, they encode exceptionally conserved *alphacoronavirus* proteins.

## 4. Discussion

This study represents one of the first coronavirus surveillance efforts targeting intestinal tissue from bats in Alabama, a region where local viral diversity remains largely uncharacterized. Extensive genetic variation and high polymorphisms make a single, universal primer set prone to false negatives [29,30,31]. We successfully implemented and validated a tiered, three-assay SYBR Green real-time PCR strategy. By pairing broad-spectrum *Alpha*-/and *Betacoronavirus* primers with highly targeted, lineage-specific *Sarbecovirus* and *Merbecovirus* assays, this platform simultaneously expands the capacity to catch divergent lineages while screening for the potential of high-priority zoonotic pathogens. Crucially, this robust, multi-assay architecture is designed to be highly accessible and cost-effective, making it a practical framework that can be readily adopted by other laboratories for regional wildlife surveillance and early pandemic preparedness.

We detected coronavirus RNA in 6 of 170 specimens, yielding an overall prevalence of 3.5%. This rate is slightly lower than or comparable to several North American surveillance indices. For instance, a pan-coronavirus screening across the Midwest identified an *Alphacoronavirus* in 6.9% of bat samples using nested RT-PCR [36]. Conversely, some studies using RT-PCR or metagenomics have reported zero detections [37]. The majority of positive detections were observed in *E. fuscus* (4/6; 66.7%), with single positive cases identified in *T. brasiliensis* and *Myotis* sp., a host distribution notably consistent with earlier Rocky Mountain surveillance, which similarly reported coronavirus detection in both *E. fuscus* (17%) and *Myotis occultus* (50%) [15], reinforcing the role of these bat genera as recurring coronavirus hosts across geographically distinct North American populations. The comparatively lower prevalence observed in this cohort likely stems from geographic variation, sampling seasons, or postmortem tissue autolysis rather than active shedding matrices in fresh fecal material or rectal swabs [38,39].

Epidemiologically, it is highly noteworthy that all six positive detections were captured exclusively via the broad-spectrum *Alpha*-/and *Betacoronavirus* assay, while the *Sarbecovirus*- and *Merbecovirus*-specific platforms yielded no positives. While bats are the evolutionary origins of the high-consequence betacoronaviruses responsible for the SARS and MERS outbreaks [40], our negative data indicate that the highly zoonotic lineages currently of greatest public health concern are not actively circulating within this Alabama cohort. This absence of high-risk *Betacoronavirus* is reassuring from a public health perspective, though it underscores that wildlife monitoring must remain vigilant for other co-circulating strains possessing unknown zoonotic potential.

The high proportion of positive detections within *E. fuscus* (4/6; 66.7%) aligns with its status as one of the most widespread, synanthropic bat species in North America. Because *E. fuscus* routinely roosts in human-occupied structures, its high viral prevalence reinforces a plausible One Health exposure pathway via contact with corporate or residential bat excreta [41,42,43,44,45]. Although direct transmission dynamics were not evaluated, the proximity of *E. fuscus* to human dwellings marks it as a high-priority host for continuous monitoring. The ability to sample fecal swabs offers a non-lethal testing alternative.

Additionally, the detection of *Alphacoronavirus* RNA in *Tadarida brasiliensis* mirrors prior southeastern surveillance, where conspecifics in Florida shed sequences matching South American strains [46]. Our single detection within a *Myotis* spp. further expands the known geographic boundary of coronavirus circulation for this ecologically vital genus into the state of Alabama [15,16,47].

There are distinct, notable limitations to this study. First, sample collection was opportunistic, tethered to public health rabies submissions, which naturally introduces biases regarding species composition, seasons, and urban localities, favoring peridomestic bats over cave-obligate or other species. Second, carcass transit delays likely induced postmortem autolysis, particularly within enzyme-dense intestinal tissues, which may have compromised RNA integrity and artificially lowered overall prevalence metrics. In addition, we focused only on intestinal tissue.

We recognize that physiological and environmental factors, including pregnancy, seasonality, and hibernation cycles, can significantly influence coronavirus shedding and prevalence in bat populations [48,49]. Due to dataset constraints, namely, a localized sample from Alabama lacking detailed demographic and seasonal monitoring, these factors could not be explicitly evaluated in our current analysis. Future surveillance efforts spanning multiple seasons and collecting granular host metadata will be crucial to clarify these temporal detection patterns.

Genetically, our BLAST and phylogenetic analyses revealed substantial nucleotide distances, with similarities ranging from 82% to 95% against known entries. This strong drop in nucleotide similarity highlights the presence of highly distinct, divergent *Alphacoronavirus* lineages circulating locally. Our diagnostic approach targets a conserved 171 bp region of the replicase gene, yielding limited phylogenetic resolution that precludes definitive taxonomic classification or evolutionary inference. Thus, the observed nucleotide divergence must be interpreted with caution, as it may indicate geographically distinct variants or uncharacterized lineages rather than novel species. These comparative constraints are compounded by a scarcity of native North American bat coronavirus sequences in public databases. Broader genomic targeting or whole-genome sequencing will be necessary to resolve the phylogenetic placement and taxonomy of these lineages.

While limited to large intestine tissue, this project confirms the presence of coronavirus RNA within the gastrointestinal tract of regional bat populations. Archiving additional tissue matrices from these carcasses will enable future multi-tissue analyses to establish viral tissue tropism and determine whether other sites, such as the respiratory tract, harbor equal or higher viral loads.

In conclusion, this work confirms active *Alphacoronavirus* circulation among multiple bat species throughout Alabama, suggesting *E. fuscus* as a major reservoir. The detected viruses appear to represent endogenous bat coronaviruses without recognized zoonotic affiliation; however, this does not diminish the importance of ongoing surveillance, as ancestral bat coronaviruses with similar characteristics have previously served as progenitors of zoonotic spillover events. By deploying an accessible, tiered multi-PCR surveillance strategy, this study provides vital baseline molecular epidemiological data for a previously undersampled region of the southeastern United States and underscores the critical need for expanded genomic characterization to decode full-length viral architectures, track long-term host–virus co-evolution, and strengthen One Health preparedness against future wildlife-origin coronavirus spillovers.

## Figures and Tables

**Table 1:** Oligonucleotides used in this study.

Targets of PCRs	Oligonucleotide Sequences (5′-3′)		Gene Target & Amplicon Size
PCR-1: *Alphacoronavirus* & *Betacoronavirus*	forward primer	GGYTGGGAYTAYCCTAAMTGTGA	RNA-dependent RNA polymerase; 247 to 294 bp
	reverse primer 1	GACANATRTTAAACACACTATTRGCATA	
	reverse primer 2	AAAGACAGARTTSGCATAAGCAGT	
PCR-2: *Sarbecovirus*	forward primer	ACAGGTACGTTAATAGTTAATAGCGTT	Envelope; 107 bp
	reverse primer	ATATTGCAGCAGTACGCACACA	
PCR-3: *Merbecovirus*	forward primer	TGWTYTTAACGAACTTAAATAAWAGCC	ORF1ab/Genomic RNA; 203 bp
	reverse primer	CGRATTGCCACGCACC	

**Table 2:** Summary of coronavirus-positive samples in this work.

Sample ID	Bat Species	County in AL	Similarities Based on BLAST (100% Query Coverage)			
			Per Nucleotide	Accession Number	Per Amino Acid	Accession Number
ID-06	*Tadarida brasiliensis*	Coosa	83.7% to 79.2%	OR725654*; OQ175650; OR725655; JQ731797; MG192609; MK569435	98.3% to 72.0%	ASL05100; AZF86117; UBB42460; ANF29193
ID-51	*Eptesicus fuscus*	Cherokee	95.3% to 93.0%	PQ554525; OL415261; OQ175395; MZ836058; OQ175397	93.0% to 66.7%	UUA80797; QCG74760; BAT21354; AKK52590
ID-145	*Eptesicus fuscus*	Etowah	84.2% to 79.1%	OQ175650; OR725654; JQ731797; PV125526; MG193609; MK569436	100% to 73.7%	ASL05100; XLE37849; AGW21696; YP009047202
ID-147	*Eptesicus fuscus*	Madison	84.3% to 78.5%	OR725654; OR725655; JQ731797; JQ731793; MG193609; MK569435	98.3% to 72.0%	ASL05100; XNJ64832; WCC61992; ANI69866
ID-169	*Eptesicus fuscus*	Jefferson	84.2% to 79.1%	OQ175650; OQ175652; OR805820; JQ731794; MG193609; MK569435	100% to 73.7%	ASL05100; ABB77038; WPV62919; XKT23283; AUF73129; ASU90382
ID-175	*Myotis* spp.	Lauderdale	84.2% to 79.1%	OQ175650; OQ175652; OR805820; JQ731794; MG193609; MK569435	100% to 70.1%	ASL05100; QGA88147; WWV93386; ASU90679; UZH24421

* Representative GenBank accession numbers of coronavirus strains.

## Data Availability

All data related to this work are included in this manuscript, including the Supplementary Files.

## Supplementary Materials

The following supporting information can be downloaded at: https://www.mdpi.com/article/10.3390/v18080887/s1, Figure S1. Primer binding sites for the *Merbecovirus* PCR assay. Multiple sequence alignment of representative subgenus *Merbecovirus* sequences illustrating the target region within the ORF1ab/genomic RNA. Red boxes indicate the binding sites for the forward primer (5′-TGWTYTTAACGAACTTAAATAAWAGCC-3′) and reverse primer (5′-CGRATTGCCACGCACC-3′), yielding an expected amplicon size of 203 bp. Figure S2. Primer binding sites for the *Sarbecovirus* PCR assay. Multiple sequence alignment of representative subgenus *Sarbecovirus* sequences targeting the envelope (E) gene region. Red boxes denote the binding sites for the forward primer (5′-ACAGGTA CGTTAATAGTTAATAGCGTT-3′) and reverse primer (5′-ATATTGCAGCAGTACGCACACA-3′), generating an expected 107 bp amplicon. Figure S3. Primer binding sites for the pan-*Alphacoronavirus* and *Betacoronavirus* PCR assay. Multiple sequence alignment of representative *Alphacoronavirus* and *Betacoronavirus* sequences within the RNA-dependent RNA polymerase (RdRp) gene. Red boxes denote the positions of the forward primer (5′-GGYTGGGAYTAYCCTAAMTGTGA-3′) and reverse primer-1 (5′-GACANATRTTAAACACACTATTRGCATA-3′), while the blue box highlights the alternative binding site for reverse primer-2 (5′-AAAGACAGARTTSGCATAAG CAGT-3′). The assay generates target amplicons ranging from 247 to 294 bp.
