# Supplementary material for: Divergent Alphacoronavirus Lineages Identified in Alabama Bats, USA
Source: Viruses. 2026 Aug 12;18(8):887. doi: 10.3390/v18080887 (PMC13517764; doi:10.3390/v18080887)
Supplement: Supplementary file 1 [file viruses-18-00887-s001.zip › viruses-4457161-supplementary.pdf]

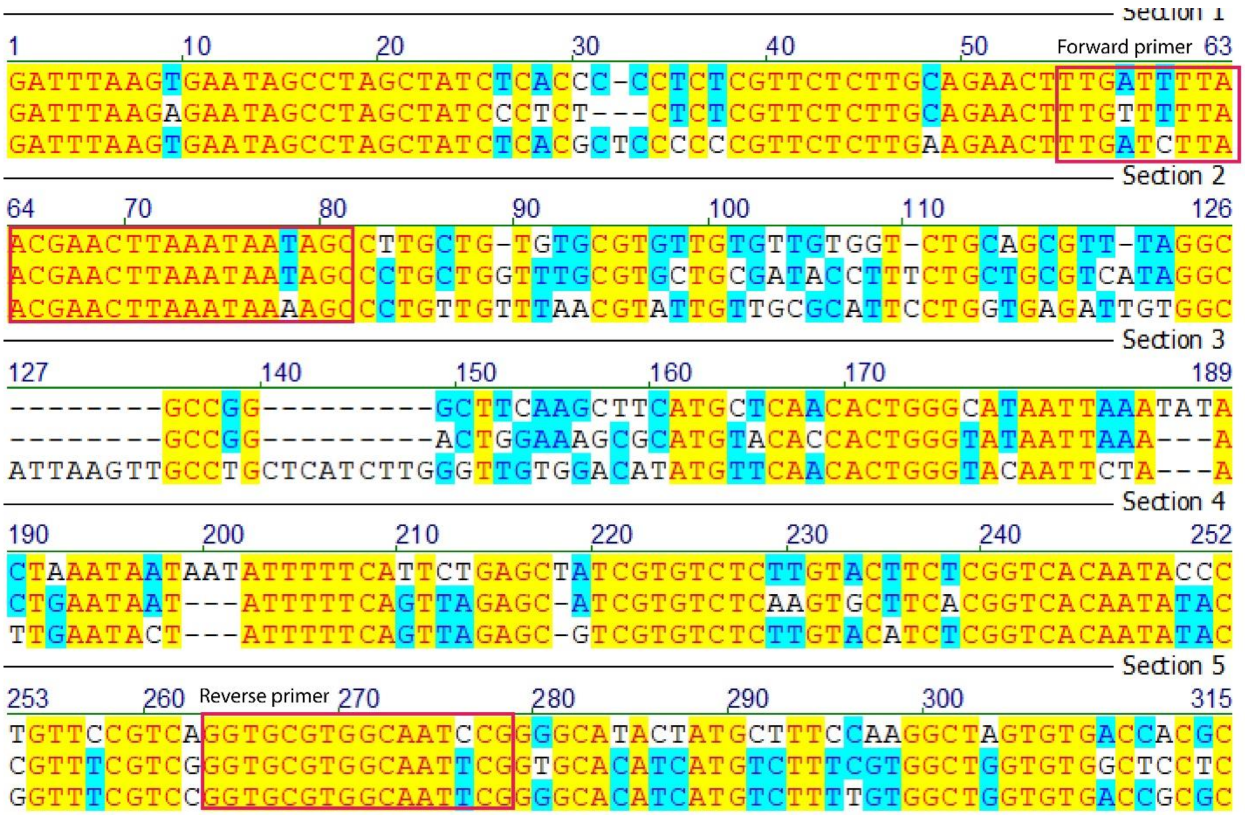

Supplementary Figure S1. Primer binding sites for the *Merbecovirus* PCR assay. Multiple sequence alignment of representative subgenus *Merbecovirus* sequences illustrating the target region within the ORF1ab/genomic RNA. Red boxes indicate the binding sites for the forward primer (5′-TGWTYTTAACGAACTTAAATAAWAGCC-3′) and reverse primer (5′-CGRATTGCCACGCACC-3′), yielding an expected amplicon size of 203 bp.

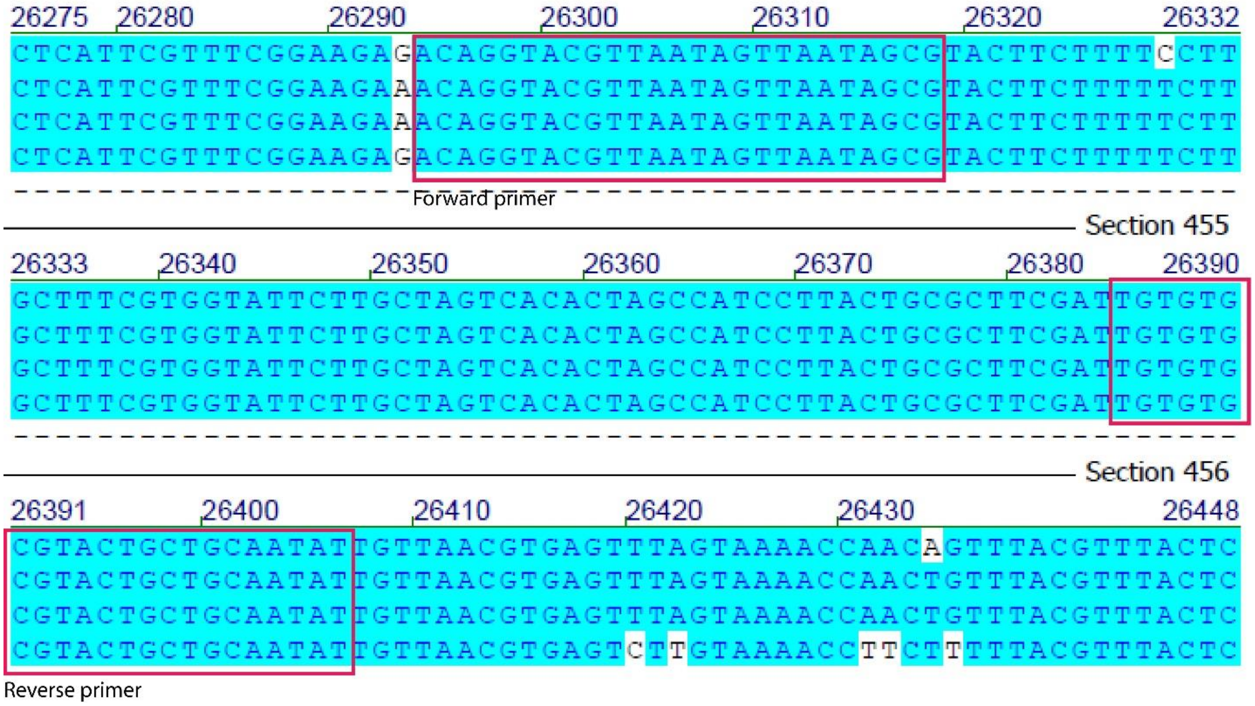

Supplementary Figure S2. Primer binding sites for the *Sarbecovirus* PCR assay. Multiple sequence alignment of representative subgenus *Sarbecovirus* sequences targeting the envelope (E) gene region. Red boxes denote the binding sites for the forward primer (5′-ACAGGTA CGTTAATAGTTAATAGCGTT-3′) and reverse primer (5′-ATATTGCAGCAGTACGCACACA-3′), generating an expected 107 bp amplicon.

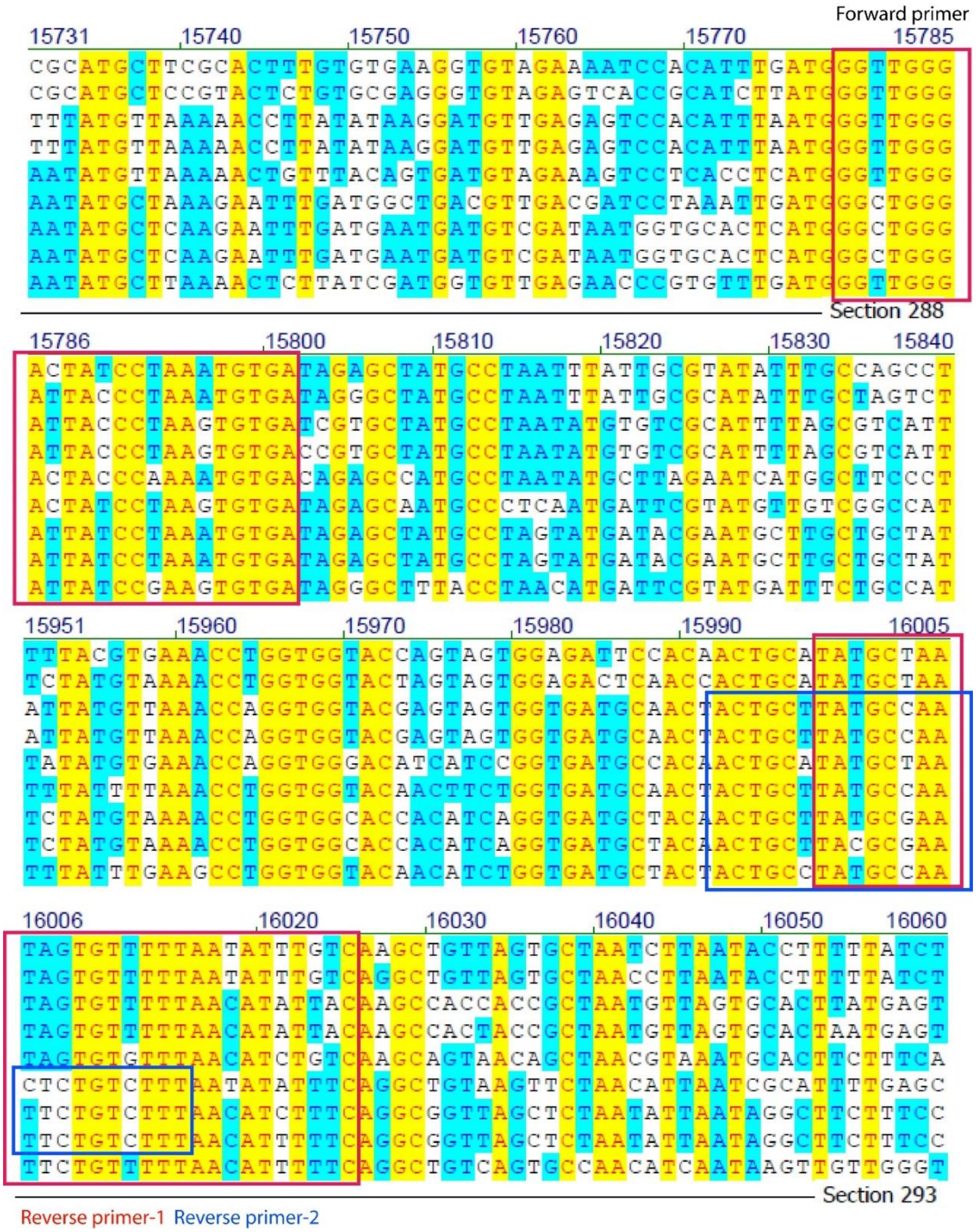

Supplementary Figure S3. Primer binding sites for the pan-*Alphacoronavirus* and *Betacoronavirus* PCR assay. Multiple sequence alignment of representative *Alphacoronavirus* and *Betacoronavirus* sequences within the RNA-dependent RNA polymerase (RdRp) gene. Red boxes denote the positions of the forward primer (5′-GGYTGGGAYTAYCCTAAMTGTGA-3′) and reverse primer-1 (5′-GACANATRTTAAACACACTATTRGCATA-3′), while the blue box highlights the alternative binding site for reverse primer-2 (5′-AAAGACAGARTTSGCATAAG CAGT-3′). The assay generates target amplicons ranging from 247 to 294 bp.
